# Immunity in Society: Diverse Solutions to Common Problems

**DOI:** 10.1371/journal.pbio.1001297

**Published:** 2012-04-03

**Authors:** Simon A. Babayan, David S. Schneider

**Affiliations:** 1 Centre for Immunity, Infection and Evolution and Institute of Immunology and Infection Research, University of Edinburgh, Edinburgh, Scotland, United Kingdom; 2 Department of Microbiology and Immunology, Stanford University, Stanford, California, United States of America

## Abstract

Understanding how organisms fight infection has been a central focus of scientific research and medicine for the past couple of centuries, and a perennial object of trial and error by humans trying to mitigate the burden of disease. Vaccination success relies upon the exposure of susceptible individuals to pathogen constituents that do not cause (excessive) pathology and that elicit specific immune memory. Mass vaccination allows us to study how immunity operates at the group level; denser populations are more prone to transmitting disease between individuals, but once a critical proportion of the population becomes immune, “herd immunity” emerges. In social species, the combination of behavioural control of infection—e.g., segregation of sick individuals, disposal of the dead, quality assessment of food and water—and aggregation of immune individuals can protect non-immune members from disease. While immune specificity and memory are well understood to underpin immunisation in vertebrates, it has been somewhat surprising to find similar phenomena in invertebrates, which lack the vertebrate molecular mechanisms deemed necessary for immunisation. Indeed, reports showing alternative forms of immune memory are accumulating in invertebrates. In this issue of *PLoS Biology*, Konrad et al. present an example of fungus-specific immune responses in social ants that lead to the active immunisation of nestmates by infected individuals. These findings join others in showing how organisms evolved diverse mechanisms that fulfil common functions, namely the discrimination between pathogens, the transfer of immunity between related individuals, and the group-level benefits of immunisation.

## Abstract 2

How do social animals, from insects to humans, limit the spread of disease by deploying community-level responses to pathogens? Active immunization of healthy ants by infected ants is one intriguing example.

It is inadvertently affirmed in the Christian countries of Europe that the English are fools and madmen. Fools, because they give their children the small-pox to prevent their catching it; and madmen, because they wantonly communicate a certain and dreadful distemper to their children, merely to prevent an uncertain evil. The English, on the other side, call the rest of the Europeans cowardly and unnatural. Cowardly, because they are afraid of putting their children to a little pain; unnatural, because they expose them to die one time or other of the small-pox.

—Voltaire (1694–1778), *Lettres Philosophiques*, Lettre XI.

All animals face the problem of preventing pathogen growth while maintaining beneficial microbiota on their most fragile epithelium. However, it is still poorly understood how organisms solve this conundrum. Animals depend upon diverse mechanisms that discriminate between self and non-self, respond to tissue damage, contain and eliminate non-self, and heal damaged tissues. While these functions are widely shared amongst animals, studying them in diverse organisms can sometimes reveal functions that weren't easily accessible in our standard models. For example, work on insects revealed a molecular mechanism that was easier to dissect initially in an insect than in a mouse (Toll signalling), thereby revolutionising our understanding of immunity in insects as well as in vertebrates, including humans. Insects also teach us about the properties of the system in a mechanism-independent manner, for example, how alternative adaptive immunity and community-level immunity functions. A desirable outcome of comparing the immune systems of diverse animal taxa is to identify common properties across the molecular, the organismal, and the population levels. Such commonalities may be the result of homology (common ancestry, e.g., pattern recognition receptors) and of convergence (similar traits acquired in different lineages, e.g., immune memory), and in some cases lead to the emergence [1] of similar system-level properties (e.g., herd immunity). It is expected that by better understanding these different levels of integration across species, one may increasingly be able to use one experimental system to inform another less amenable to scientific enquiry.

Descriptions of local reactions to foreign bodies in starfish larvae marked the birth of cellular immunology [2]. Upon infection, invertebrates expand relatively poorly defined populations of immune cells that are capable of phagocytosis and encapsulation, or can produce an acellular coat of melanin to encapsulate microbes [3],[4], and increase their secretion of antibacterial and antifungal peptides [5],[6]. The mechanisms governing the response to microbial products are highly conserved, and research on insects has often led the way in discovering these processes. For example, the discovery of antimicrobial peptides (AMPs) in the moth *Cecropia* [7] led to the analysis of the genes for these AMPs, the identification of promoters, the implication of NFκB signalling, and the finding that Toll activation triggers this response [8].

Homologues of Toll are a central molecular family in vertebrate immune signalling. Medzhitov et al. first showed that a Toll-like receptor (TLR) family member could activate immune cells [9], and Poltorak et al. soon after demonstrated that TLR-4 was required to respond to a major component of bacterial membranes, LPS [10]. One trait that all of these receptors have in common is that they are encoded in the germline, and expressed and inherited with little to no modification from one generation to the next. These receptors have been selected to recognise common molecular patterns present on pathogens. Pattern recognition receptors on dendritic cells and other antigen presenting cells trigger inflammation and antigen processing and presentation to specialised cells (T lymphocytes), thereby engaging adaptive immunity through somatic gene rearrangement in lymphocytes and the commitment of a subset of those cells for future encounters with the same molecular patterns—i.e., immune memory.

Historically, humans have taken medical advantage of immune memory: inoculating small doses of *Leishmania* in a chosen area of the body (leishmanisation) protects against more severe pathology caused by natural exposure; Edward Jenner (1749–1823) used exposure to cowpox to cross-protect humans against the more virulent smallpox, thereby inventing vaccination; Louis Pasteur (1822–1895) discovered that by artificially weakening (attenuating) a pathogen or by extracting adequate innocuous elements, one could reduce or eliminate side effects of the immunisation and protect against infection following natural exposure. All vaccines to this day are based on those early discoveries.

The distinction between innate and adaptive immunity is blurry. In vertebrates, adaptive immunity requires sustained “innate” activation, as shown by the importance of adjuvants in vaccine formulations. Insects lack B, T, and dendritic cells and thus cannot raise the sort of adaptive immune responses found in vertebrates, but that doesn't mean that they can't fine-tune or enhance their responses to secondary infection. Invertebrates have been reported to introduce somatic diversity in gene structure [11], and they can take advantage of alternative splicing of complex genes to generate transcripts that provide more specificity than would be predicted from the “innate” genome [12]–[15]. Insects have been found to have a variety of “memory” responses where an individual insect can enhance its immune response upon secondary exposure to a pathogen [13],[15]. Immune responses have even been found to have some cross-generational properties, where exposure of a father or mother insect to a microbe will alter the offspring's ability to deal with that microbe [16]. Such properties are now being found in vertebrate immune systems that were originally thought to be purely innate [17] and may be leading to the birth of a new field called “trained immunity” [18].

Beyond the survival of the host, immune responses affect the survival and transmission of pathogens. Thus, the structure of a population of hosts (frequency of contact, density, etc.) affects the spread and persistence of diseases [19]. Social species in particular are characterised by high population densities with high contact frequencies, which increase the chances of transmission.

However, when a sufficiently high proportion of individuals within a population becomes immune (either through prior exposure or through mass vaccination), community or “herd” immunity emerges [20], whereby individuals that are poorly immunised are protected by the collective “immune firewall” provided by immunised neighbours (Figure 1). In humans and other vertebrate communities, the mechanisms involved rely heavily on adaptive immunity and immune memory: responses to a previously encountered pathogen are faster and stronger than those to a novel pathogen, and thus individuals are better at blocking its spread. Herd immunity is but one of the defences social animals generate against disease—waste management, food quality assessment, and containment of sick individuals are some of the behavioural traits that help to reduce exposure to pathogens within social groups.

**Figure 1:**
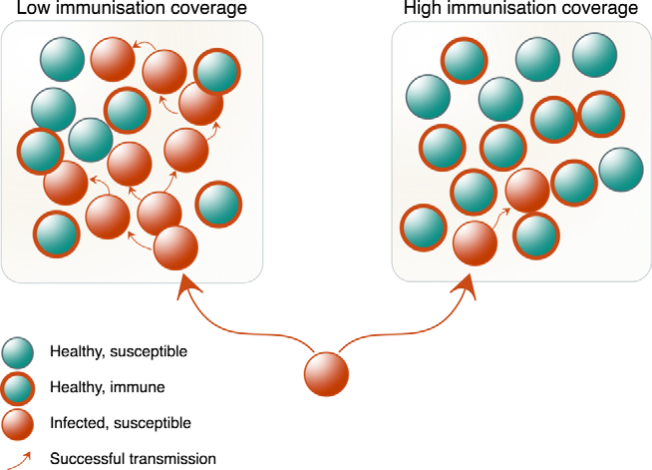
The double-edged sword of social life. When a sufficiently high proportion of a population is immune to a pathogen, transmission to non- and poorly immunised individuals (e.g., the young, the immunocompromised, and the weak responders to immunisation) is blocked by the barrier formed by immunised individuals. Conversely, when immunisation rate is insufficient, non-immunised individuals are at greater risk of becoming infected during social contacts.

Yet, insects also form societies, and evidence is accumulating that they manage waste [21], maintain food quality [22], and remove dead individuals [23], and that they can modulate their behaviour and immune responses when exposed to pathogens and transfer that immunity between individuals. Termites exposed to high doses of fungal conidia transmit vibratory alarm signals through the substrate on which they stand. Their nestmates flee in response, thus reducing their risk of infection [24]. Social immunity often involves direct physical contact, with inducible mutual grooming being a powerful way to limit the contagion from exposed individuals. The ingestion of fungal spores by the subterranean termite *Reticulitermes flavipes* prevents their germination, so that grooming and even cannibalism of spore-exposed termites effectively stops disease transmission in the colony [25]. One possible mechanism for the inhibition of pathogen growth was reported in honey bees to be the competition from non-pathogenic gut commensals [26],[27]. A step further in colony-level disease defence is the direct immunisation of uninfected nestmates by exposed individuals. One route social insects use to transfer immunity is trophallaxis—the sharing of nutrients and fluids between individuals via mouth-to-mouth or anus-to-mouth—during which antimicrobial activity, likely carried by AMPs in the saliva [28], may be exchanged [29],[30]. It is inferred that infected individuals increase the amounts of AMPs present in the regurgitate and thereby increase group-level immunity through passive transfer of immune factors, i.e., the recipient is immunised without having to mount an immune response itself.

In this issue of *PLoS Biology*, Konrad et al. bring a clearer understanding of another mechanism of social immunisation, through which group-level antifungal immunity may emerge. They covered *Lasius neglectus* ants with lethal doses of the entomopathogenic fungus *Metarhizium anisopliae*, and those ants were then allowed to interact with nestmates. In so doing, they exposed their nestmates to fungal doses that were too low to induce death (except for 2% that died of the infection), but that were sufficient to induce a specific pattern of anti-fungal immune gene expression. As a result, the recipients of the inoculum were less likely to die from a subsequent lethal dose of the same microbe, and mathematical modelling suggests that these responses would allow colonies to recover more rapidly. As first suggested by Rosengaus and Traniello [24], these phenomena are strongly reminiscent of variolation as practised by humans, whereby exposure to controlled low doses of a pathogen protects individuals against future infections. Unlike vaccination however, the fungal spores transmitted in the system studied by Konrad et al. did not appear to be attenuated, for example, by digestive enzymes, and remained infective. The authors used a combination of approaches to identify the mechanisms underlying social immunisation in ant colonies: mathematical modelling, and behavioural, microbiological, immunological, and molecular techniques, which, taken together, offer an exciting *proof-of-concept* that group-level immunity may be experimentally manipulated and modelled. How this relates to animal and human epidemiology still needs to be assessed, but it is very likely that sound evolutionary inferences may readily be made from such studies.

With regard to the immune mechanisms and dynamics that operate in social insect groups, and specifically with regard to the studies presented by Konrad et al., it would be fruitful to examine, for instance, the cellular basis of the immune specificity suggested by gene expression patterns; whether prior exposure enables more rapid and/or stronger responses to lower doses of pathogen; how much cross-protection against other pathogens is thus generated; and whether insect social immunisation persists only as long as individuals are exposed to the pathogen or whether immune memory can produce long-term social immunisation in invertebrates.

Social immune mechanisms are unlikely to be conserved at a molecular level between insects and mammals; but rather, social systems will have independently evolved their own solutions. Thus, in tightly-knit groups like those formed by social species, the additive effect of individual sanitary responses, be they immune or behavioural, generates a “communal immune system” leading to the mitigation of pathogen transmission. By studying social immunity at a system level in insects, perhaps we can find emergent properties that we have been missing in another important social animal—the human.
